# HDAC11: A novel inflammatory biomarker in Huntington’s disease

**DOI:** 10.17179/excli2022-4741

**Published:** 2022-03-24

**Authors:** Vishal Kumar, Simranjit Kaur, Lakshay Kapil, Charan Singh, Arti Singh

**Affiliations:** 1 ISF College of Pharmacy, Department of Pharmacology, Moga-142001, Punjab, India, affiliated to IK Gujral Punjab Technical University, Jalandhar, Punjab, India; 2 ISF College of Pharmacy, Department of Pharmaceutics, Moga-142001, Punjab, India, affiliated to IK Gujral Punjab Technical University, Jalandhar, Punjab, India

## ⁯

Huntington's disease (HD) is an inherited fatal neurodegenerative disorder associated with striatal-specific GABAergic medium-spiny neurons (MSNs) characterized by choreiform movements, psychiatric, cognitive, and motor dysfunctions. The average HD prevalence is 5 in 100,000 people in the western world; small frequencies are known in Africa, Japan, China, and Finland. HD is caused by the CAG (cytosine, adenine, guanine) repeated expansion in exon-1 of the Huntington (Htt) gene. CAG triplet repeats in exon-1 are responsible for the increase in the polyglutamine (poly Q) on Htt in HD patients. Normal Htt has 35 CAG repeats, whereas mutant Huntington (mHtt) > 40 CAGs, patients with 36-39 CAG repeat extensions are at risk of HD. The exact mechanism of HD is still unknown; however, neuroinflammation, mitochondrial dysfunction, oxidative stress, excitotoxicity, loss of brain-derived neurotrophic factor (BDNF), and apoptosis are well-accepted mechanisms in HD. There is no effective therapeutics for the treatment of HD. The available therapies focus only on psychiatric and neurological symptoms to improve quality of life (Kumar and Singh, 2021[5]). 

Neuroinflammation is defined as an inflammatory response or activation of immune responses within the CNS (central nervous system), particularly in the brain and spinal cord, which is triggered by chemokines (CCL2, CCL5, CXCL1), cytokines (IL-1, IL-6, TNF-α), secondary messengers (prostaglandins and NO) and reactive oxygen species (ROS) (Kumar et al., 2021[6]). However, neuroinflammation may lead to psychological, immunological, biochemical, and physiological consequences. It is reported that neuroinflammation contributes to the pathogenesis of various brain disorders such as Alzheimer's disease (AD), Parkinson's disease (PD), Huntington's disease (HD) (Kumar et al., 2021[6]), multiple sclerosis (MS), amyotrophic lateral sclerosis (ALS) and traumatic brain injury (TBI) (Guzman-Martinez et al., 2019[2]; Kumar et al., 2021[6]).

Histones are water-soluble proteins wrapped around DNA. The interaction between DNA and histones is regulated by the following enzymes, e.g., histone acetyltransferases (HATs) and histone deacetylases (HDACs). So far, 18 subtypes of HDACs have been discovered in mammals, which are divided into four categories according to their catalytic domains: class I HDACs consist of HDAC1, 2, 3, and 8, class II HDACs are further divided into two types, class IIa (HDAC 4, 5, 7 and 9) and Class IIb (HDAC6 and 10). Class III HDACs are also known as Sirtuins (1-7), while Class IV is HDAC 11. HDAC11 is the smallest member of the HDAC family, with more than 80 % of its 347 amino acid sequences allocated to its catalytic domain with minimal N- and C-terminal elongation, i.e., free of predicted protein binding sites (Kumar et al., 2021[4]).

HDAC11 was first discovered by Lin Gao and colleagues in 2002. Human HDAC11 is a more conserved protein, identical to mouse (91 %), Arabidopsis (51 %), and Drosophila (52 %). HDAC11 is particularly expressed in the brain, skeletal muscle, kidney, heart, and testis. It is distributed mainly in both the cytoplasm and nucleus of mature neurons, human T cells, retinal ganglion cells, and macrophages. The study found that the highest expression of HDAC11 was observed in the granule cell layer and Purkinje neurons in the adult rat brain, leading to the conclusion that HDAC11 plays a significant role in ataxia syndrome (lack of coordination of voluntary movements) and locomotor activity. In addition, it is also expressed in the hippocampus, suggesting that it may affect learning and memory. However, the expression of HDAC11 in the hippocampus regulates neuronal maturation via control of the complexity and length of neuron ends (Kumar et al., 2021[4]).

Interleukin-10 is a cytokine that has anti-inflammatory effects based on its ability to regulate macrophage and dendritic cell function, along with the production of pro-inflammatory cytokines (O'Garra and Vieira, 2007[8]). The inactivation of macrophages and DCs, as well as the reduction of pro-inflammatory cytokine secretion, MHC class II, and co-stimulatory molecule expression, are part of the IL-10 suppressive effects. IL-10 reduces the maturation of DCs from monocyte progenitors and the ability of macrophages to destroy intracellular pathogens by reducing TNF synthesis. IL-10-mediated signal transducers and activators of transcription 3 (STAT3) regulate to some extent a variety of genes in these cells, although the mechanism behind the IL-10. The study showed that colonization of the gut with specific microbes causes inflammatory bowel disease in IL-10 knockout mice, but genetic testing in humans has demonstrated the importance of IL-10 in preventing harmful inflammation in the gut (Allavena et al., 1998[1]). In addition, IL-10 has been shown to inhibit apoptosis by activating the phosphatidylinositol-4,5-bisphosphate-3-kinase (PI3K)/Akt cascade and inhibiting the production of anti-apoptotic proteins such as Bcl-2 and Bcl-xl leading to downregulation of caspase-3 expression. In addition, IL-10 stimulates the synthesis of transforming growth factor (TGF) - by astrocytes. IL-10 receptor signaling has been implicated in enhanced cellular survival and regulation of adult neurogenesis in neurons. Microglial cells are the most studied innate immune cells of the brain and consequently the most studied cytokine producers, including IL-10. The *in vitro* study showed that TLR activation is involved in IL-10 synthesis in microglial cells. Microglial cells produced IL-10 in response to activation of TLR 2, 3, 4, and 9. The production of IL-10 by TLR-activated microglia can be further controlled via other molecules. This is the case with adenosine, which stimulates TLR2 and 4 to increase the synthesis of IL-10 in microglial cells while suppressing the synthesis of pro-inflammatory cytokines (Jack et al., 2005[3]). 

T cell activation and tolerance are both induced by antigen-presenting cells (APCs). IL-10, an immunosuppressive cytokine, is important in the formation of tolerogenic APCs and in preventing autologous tissue injury. Negative and positive feedback loops involving numerous transcriptional regulators and cell type-specific signaling pathways determine a key regulatory mechanism for IL-10 production at the transcriptional level. STAT3, Sp1, AP-1, NF-κB, C/EBP, and GATA3 are all major transcriptional regulators of IL-10, while some are required for activation of IL-10 gene transcription (STAT3, Sp1). The molecular mechanisms that control the balance between these different signaling pathways are still unknown (Saraiva and O'Garra, 2010[9]). HDACs are targeted to promoter regions by co-repressors or multiprotein transcription complexes, and they use chromatin changes to regulate gene expression. It was recently discovered that among all members of this HDAC family, the predominantly nuclear HDAC11 attracts the IL-10 gene promoter, which negatively affects its expression by activating histone deacetylation and downregulating IL-10 expression. This suppression of anti-inflammatory protein could result in increased production of inflammatory cytokines, whichever is capable to bring a storm of cytokines leading to glial cells and astrocyte activation. All these factors collectively will result in neuroinflammation, which is considered as initial hallmark of various neurodegenerative diseases. Several studies support this phenomenon that inhibition of HDAC11 can possibly be a therapeutic target for the management of various brain disorders and different cancers in which HDAC11 is found to be more expressed (up to 4 %) (Liu et al., 2020[7]) (Supplementary Figure 1).

It is widely reported that HD is a rare disease and the exact mechanism of HD is still unknown. So there is a need for more research to find the causative mechanism that can help develop a new therapeutic strategy against HD. Therefore, with support from the discussion above, we hypothesized that HDAC11 could be a novel inflammatory biomarker in HD because it directly inhibits the expression of IL-10 (acting as an anti-inflammatory marker) and leads to inflammation that continues to lead to the development of Huntington's disease. Villagra et al. (2009[10]) performed an *in vivo* study and showed that HDAC11 negatively downregulates the expression of IL-10. However, targeting HDAC11 with specific inhibitors (FT895 and SIS7 & SIS17) could bring a new revolution against HD. In the coming years, the novel drug design with HDAC inhibitors could increase its potency and specificity by overcoming the problems associated with HDAC inhibitors. Therefore, the development of specific and potent HDAC11 inhibitors could be a new therapeutic strategy against HD. We suggest that more research is needed to elucidate the exact mechanism of HDAC11-mediated Huntington's disease.

## Declaration

### Acknowledgment

We are thankful to the Institute for providing an excellent platform.

### Conflict of interest 

The authors declare there is no conflict of interest.

### Funding 

Not available.

### Authors' contributions 

Vishal Kumar wrote the review, Simranjit Kaur and Lakshay Kapil helped with the figure and references, Charan Singh helped in editing the manuscript, Arti Singh designed the layout and critically revised the manuscript. The authors declare that all data were generated in-house and that no paper mill was used. 

## Supplementary Material

Supplementary information
