# Supplementary material for: HDAC11: A novel inflammatory biomarker in Huntington’s disease
Source: EXCLI J. 2022 Mar 24;21:647–50. doi: 10.17179/excli2022-4741 (PMC9149973; doi:10.17179/excli2022-4741)
Supplement: Supplementary information [file EXCLI-21-647-s-001.pdf]

EXCLI Journal 2022;21:647-650 – ISSN 1611-2156
Received: February 03, 2022, accepted: March 16, 2022, published: March 24, 2022

Supplementary information to:

Letter to the editor:

HDAC11: A NOVEL INFLAMMATORY BIOMARKER IN HUNTINGTON'S DISEASE

Vishal Kumar[a], Simranjit Kaur[a], Lakshay Kapil[a], Charan Singh[b], Arti Singh[a*]

[a] ISF College of Pharmacy, Department of Pharmacology, Moga-142001, Punjab, India
[b] ISF College of Pharmacy, Department of Pharmaceutics, Moga-142001, Punjab, India
[a+b] affiliated to IK Gujral Punjab Technical University, Jalandhar, Punjab, India

* **Corresponding author:** Dr. Arti Singh, ISF College of Pharmacy, Department of Pharmacology, Moga-142001, Punjab, India. E-mail: artiniper@gmail.com

https://dx.doi.org/10.17179/excli2022-4741

This is an Open Access article distributed under the terms of the Creative Commons Attribution License (http://creativecommons.org/licenses/by/4.0/).

EXCLI Journal 2022;21:647-650 – ISSN 1611-2156
Received: February 03, 2022, accepted: March 16, 2022, published: March 24, 2022

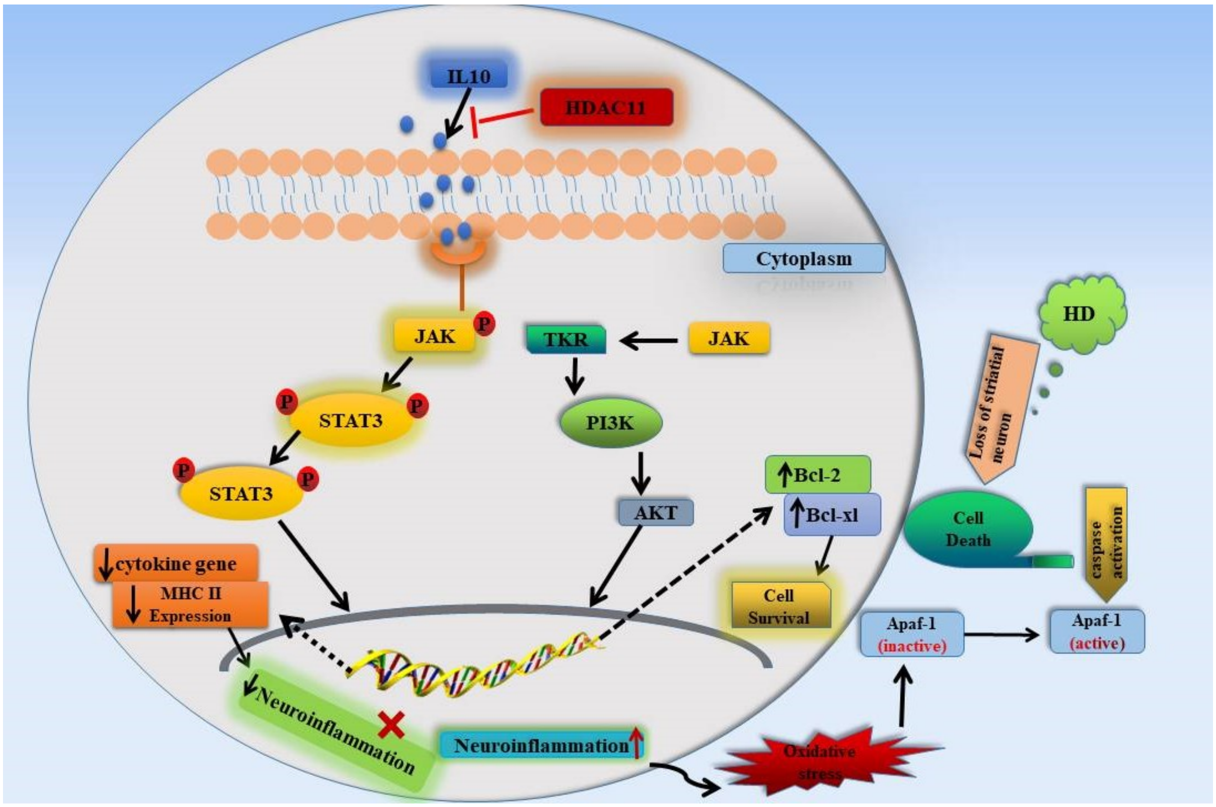

Supplementary Figure 1: Mechanistic representation of HDAC11 mediated HD pathogenesis. IL-10 acts through the JAK-STAT pathway migrates to the cell nucleus and lowers cytokine levels, resulting in the prevention of inflammation. In addition, IL-10 inhibits apoptosis and acts through the PI3K/AKT pathway to increase production of Bcl-2 and Bcl-xl by decreasing caspase-3 expression and increasing cell survival. HDAC11 acts on IL-10 and inhibits its anti-inflammatory activity, leading to increased inflammation and ROS production, which can further lead to neuronal cell death via caspase signaling pathway activation.
